# Seasonal Changes of Airborne Bacterial Communities Over Tokyo and Influence of Local Meteorology

**DOI:** 10.3389/fmicb.2019.01572

**Published:** 2019-07-16

**Authors:** Jun Uetake, Yutaka Tobo, Yasushi Uji, Thomas C. J. Hill, Paul J. DeMott, Sonia M. Kreidenweis, Ryohei Misumi

**Affiliations:** ^1^Department of Atmospheric Science, Colorado State University, Fort Collins, CO, United States; ^2^National Institute of Polar Research, Tachikawa, Japan; ^3^Department of Polar Science, School of Multidisciplinary Sciences, SOKENDAI (The Graduate University for Advanced Studies), Tachikawa, Japan; ^4^National Research Institute for Earth Science and Disaster Resilience, Storm, Flood and Landslide Research Division, Tsukuba, Japan

**Keywords:** bioaerosol, air, DNA, airborne microbiome, urban microbiome, next generation sequencing

## Abstract

In order to study airborne bacterial community dynamics over Tokyo, including fine-scale correlations between airborne microorganisms and meteorological conditions, and the influence of local versus long-range transport of microbes, air samples were collected on filters for periods ranging from 48 to 72 h. The diversity of the microbial community was assessed by next generation sequencing. Predicted source regions of airborne particles, from back trajectory analyses, changed abruptly from the Pacific Ocean to the Eurasian Continent in the beginning of October. However, the microbial community composition and the alpha and beta diversities were not affected by this shift in meteorological regime, suggesting that long-range transport from oceanic or continental sources was not the principal determinant controlling the local airborne microbiome. By contrast, we found a significant correlation between the local meteorology, especially relative humidity and wind speed, and both alpha diversity and beta diversity. Among four potential local source categories (soil, bay seawater, river, and pond), bay seawater and soil were identified as constant and predominant sources. Statistical analyses point toward humidity as the most influential meteorological factor, most likely because it is correlated with soil moisture and hence negatively correlated with the dispersal of particles from the land surface. In this study, we have demonstrated the benefits of fine-scale temporal analyses for understanding the sources and relationships with the meteorology of Tokyo’s “aerobiome.”

## Introduction

Microorganisms are abundant in the atmosphere, with numbers ranging from 10^4^–10^5^ cells m^−3^ in ambient air on mountain peaks (Bowers et al., 2012) to 10^6^–10^7^ m^−3^ in desert dust storms (Maki et al., 2017b). The atmosphere is widely recognized as a highly diverse microbiome (Franzetti et al., 2011; Bowers et al., 2011a,b, 2012, 2013; Bertolini et al., 2013; DeLeon-Rodriguez et al., 2013; Woo et al., 2013; Yooseph et al., 2013; Gandolfi et al., 2015; Seifried et al., 2015; Genitsaris et al., 2017; Šantl-Temkiv et al., 2018; Archer et al., 2019). Airborne bacteria and fungi also have the potential to cause human disease, as reviewed by Salem and Gardner (1994). For example, airborne fungi have been identified as the cause of respiratory problems such as asthma after thunderstorms (Grinn-Gofroń and Strzelczak, 2013), and also the lymph node syndrome, Kawasaki disease (Rodó et al., 2014). Endotoxins from airborne bacteria are also associated with health issues (Mueller-Annelling et al., 2004). Airborne microorganisms have impacts not only on human health, but also on climate and microbial biogeography. For example, the plant pathogen *Pseudomonas syringae* and related phylloplane bacteria have strong ice nucleation ability at 33°C warmer (−5°C) than the homogeneous freezing temperature of cloud droplets composed of pure water (Maki et al., 1974; Lindow et al., 1978; Morris et al., 2008; Hill et al., 2014). Thus, airborne ice nucleating bacteria may promote the formation of ice in clouds, which may modify cloud radiative forcing and promote precipitation. Until recently, most airborne microorganism studies depended on culture-based methods (e.g., Lindemann and Upper, 1985; Shaffer and Lighthart, 1997). However, culture-independent methods using next generation sequencing (NGS) are now widely applied (Franzetti et al., 2011; Bowers et al., 2011a,b, 2012, 2013; Bertolini et al., 2013; DeLeon-Rodriguez et al., 2013; Woo et al., 2013; Yooseph et al., 2013; Gandolfi et al., 2015; Seifried et al., 2015; Genitsaris et al., 2017; Šantl-Temkiv et al., 2018). This technology transforms our ability to describe spatial and temporal variability of many airborne microorganisms, and enables us to compare their temporal variation under different meteorological conditions. However, the relationship between aerial microbial community composition and meteorology is still not well characterized (Gunthe et al., 2016). For example, Bowers et al. (2011a) showed different land-use types, rather than local weather including relative humidity and wind speed, control the airborne bacterial composition in northern Colorado, US, during the early summer season. On the other hand, Gandolfi et al. (2015) showed wind speed and relative humidity affected airborne microbial community structure in two urban sites, in northern Italy. Šantl-Temkiv et al. (2018) showed that the alpha diversity of bacterial communities is positively correlated with air temperature and negatively correlated with relative humidity in western Greenland during mid-summer. Studies are often focused on urban areas, because these contain sources of human pathogens, such as water treatment facilities and densely populated areas. While spatial and temporal variations of bioaerosols in urban environments have been frequently studied in many cities (Brodie et al., 2007; Frohlich-Nowoisky et al., 2009; Bowers et al., 2013; Woo et al., 2013; Gandolfi et al., 2015; Genitsaris et al., 2017), few studies (Bowers et al., 2013) have used consecutive short-period samples to explore correlations between variation of the microbiome and meteorology. Additionally, samples from these studies were taken near ground level (generally several meters above) or on roof tops of lower buildings, with the result that specific local sources very near to sampling sites likely predominate over microorganisms originating regionally (e.g., Archer et al., 2019).

In this study, we investigated: (1) airborne bacterial communities over Tokyo, (2) fine-scale correlations between airborne microorganisms and meteorological conditions, and (3) the influence of local versus long-range transport of microbes, *via* analysis of a series of consecutive 48–72 h samples collected at a height of 458 m from the tallest communication/observation tower in Japan from summer 2016 to winter 2017.

## Materials and Methods

### Sampling of Air and Reference Samples

Airborne microorganisms were collected at a 458-m level measurement site (Misumi et al., 2018) on the western side of the Tokyo Skytree (Supplementary Table S1) from August 2016 to February 2017. Eight sets of 2-m-long conductive silicon tubes were fixed to the outside wall of the Tokyo Skytree with their inlets placed under a wind and rain shield, and sterilized inline NILU filter holders (Norwegian Institute for Air Research) attached to the inner ends. These were fitted with pre-combusted (500°C for 2 h) 47-mm-diameter quartz filters with 99.9% retention rate for 0.3 μm particles (Tissuquartz™ Filters, 2500 QAT-UP, Pall). The vacuum sides of the NILU filter holders were connected to low volume samplers (LV-40BW, Sibata Scientific Technology) programmed to sample from one unit at a time. Air samples were collected at a flow rate of 15–30 L min^−1^ for 48–72 h for each filter, and then the sampler switched to a new filter unit; thus, sampling was continuous throughout the study period. Sampling details are described in Supplementary Table S2. In order to avoid degradation of filter samples during and after sampling, all filter holders were placed in a 4°C refrigerator (JR-N40G, Haier) until retrieval (usually at 2–3 week intervals). Reference environmental (potential source) samples for source estimation were taken around Tokyo Skytree on Jan 3, 2017, and from around the building of the National Institute of Polar Research (NIPR) on June 6, 2016 (Supplementary Table S1, Supplementary Figure S1). We selected these sites because there are very limited open spaces in the Tokyo metropolis. Soil samples were placed in a 5-ml sterile plastic tube containing 2 ml of RNAlater (Thermo Fisher Scientific, MA, USA) using a pre-cleaned stainless-steel spoon. Seawater, river water, and pond water were sampled directly into a 50-ml sterile plastic bottle. All soil and water samples were kept at −20°C prior to DNA extraction.

### DNA Extraction, Polymerase Chain Reaction, and DNA Sequencing

In order to avoid contamination, all processes prior to the polymerase chain reaction (PCR) amplification were done in a laminar flow clean bench (PCV-1305BNG3-AG, Hitachi). The clean bench was sanitized with a UV lamp overnight, and pipettes were sterilized in a DNA cross linker (CL-1000, UVP) box inside the clean bench. Genomic DNA in bioaerosols captured on quartz filters was extracted using the FastDNA™ SPIN Kit for Soil (MP Biomedicals, Santa Ana, CA). The quartz filter was initially pulverized during the bead beating step, but in order to maximize the yield of DNA (DNA adsorbs to quartz fibers), all fragments of the filter were carried over until the final elution step. Partial 16S rRNA gene sequences, spanning the V3 and V4 regions, were amplified using the primers Bakt_341F (5´ CCTACGGGNGGCWGCAG 3′) and Bakt_805R (5´ GACTACHVGGGTATCTAATCC 3′), with Illumina overhang adaptor sequences attached to their 5′ ends, by KAPA HiFi HotStart ReadyMix (KAPA Biosystems, MA, USA). PCR conditions comprised 35 cycles of denaturation at 95°C for 30 s, annealing at 55°C for 30 s, and elongation at 72°C for 30 s and an additional final elongation at 72°C for 5 min using a GeneAmp PCR System 9700 (Applied Biosystems, CA, USA). Subsequent clean-up and indexing of PCR amplicons were performed following Illumina methods for 16S metagenomic sequencing library preparation^1^. All samples were pooled into one flow cell of a MiSeq sequencer (Illumina, San Diego, CA) and sequenced at NIPR using a MiSeq V3 reagent kit.

### Exact Sequence Variant Analysis

In order to study sequence differences in greater detail than the conventional 97% operational taxonomic unit (OTU) approach, we used a newly developed DADA2 (1.4) method which, by incorporating an error model, is able to infer sequence reads with single nucleotide resolution (Callahan et al., 2016). Since airborne bacterial DNA is relatively low in concentration and contamination during processing of samples is a known concern (e.g., Salter et al., 2014; de Goffau et al., 2018), air samples and three types of control sequences were analyzed together: (1) an extraction-PCR control using blank filters (07 W4, 14 W4, 16 W3); (2) a PCR control (Negative1, Negative2); and (3) laboratory amplicon contamination (sequences from previous studies analyzed at NIPR: DRA004099–DRA004103). Next, Amplicon Sequence Variants (ASVs) in all contamination controls were removed from further analysis. Taxonomy was assigned by the Naive Bayes Classifier method in the Ribosomal Database Project (RDP) Classifier (Wang et al., 2007) implemented in the DADA2 program against the customized Silva 128 database (data are available at https://doi.org/10.6084/m9.figshare.7772864) (Quast et al., 2013). All potential chimera sequences were removed by DADA2 and all chloroplast and mitochondria were removed manually. Alpha diversities, rarefaction curve of alpha diversities (Chao 1, the Shannon index and the reciprocal of Simpson’s index), unweighted UniFrac distances analysis (Lozupone and Knight, 2005) and unweighted Pair Group Method with Arithmetic Means (UPGMA) were analyzed by QIIME (Caporaso et al., 2012). Chao 1 estimates the total ASV richness; the Shannon index is a general diversity measure that is positively correlated with both diversity and evenness, being sensitive to differences in abundance of rare ASVs; and Simpson’s reciprocal is a measure of evenness, which has a lower bound of 1 for a community composed of only one ASV and, for example, a value of 10 for a community containing 10 equally abundant ASVs (Hill et al., 2003). A phylogenetic tree for unweighted UniFrac distances analysis was constructed using FastTree (Price et al., 2009) implemented in Geneious software, version 10.0.9^2^. Based on rarefaction curve results and number of filtered sequences, sequences were rarefied to 970 for alpha diversity and 870 for beta diversity estimation. All codes and data are available from https://github.com/JUetake/TokyoSkytree-Bacteria. A taxonomy heatmap was created by the R Package “superheat” (Barter and Yu, 2018).

### Source Estimation

“SourceTracker” is a Bayesian approach program to estimate the proportion of exogenous sequences in a given community that come from possible source environments (Knights et al., 2011). We used the latest version, SourceTracker2^3^, for source estimation of sampled bioaerosols sampled. Twenty-three reference samples collected around Tokyo Skytree (eight sites: Supplementary Figure S1) were analyzed as possible sources of airborne bacteria. In order to focus on local bacteria around the Tokyo area, six Amplicon Sequence Variants (ASVs), which have significant differences (*p* < 0.001, Mann-Whitney) between Pacific and continental periods (explained in section “Results and Discussion”), were removed from analysis.

### Statistical Data Analysis

Correlations between alpha diversities, source contributions, and meteorological data were analyzed by Spearman’s correlation using the R package “ggcorrplot.” Mann-Whitney U tests between Pacific and continental periods were performed with XLSTAT software^4^. Analysis of similarities (ANOSIM) and permutational multivariate analysis of variance (PERMANOVA) for unweighted UniFrac dissimilarity matrices were performed using PRIMER 7 (Plymouth, UK).

### Meteorological Data

**Figure 1:**
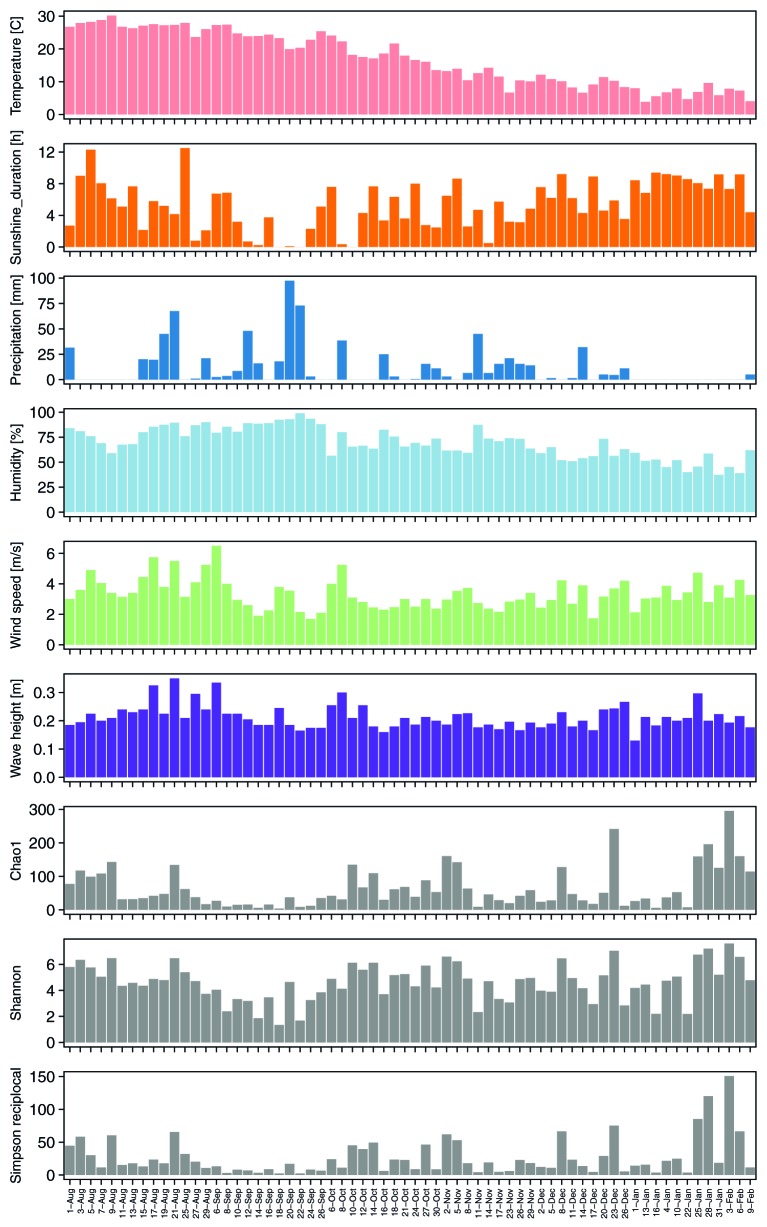
Seasonal changes of meteorological factors (temperature: pink, sunshine duration: orange, precipitation: blue, humidity: light blue, and wind speed: light green) from nearest automatic weather stations, wave height (purple), and alpha diversities (gray) during sampling periods.

**Figure 2:**
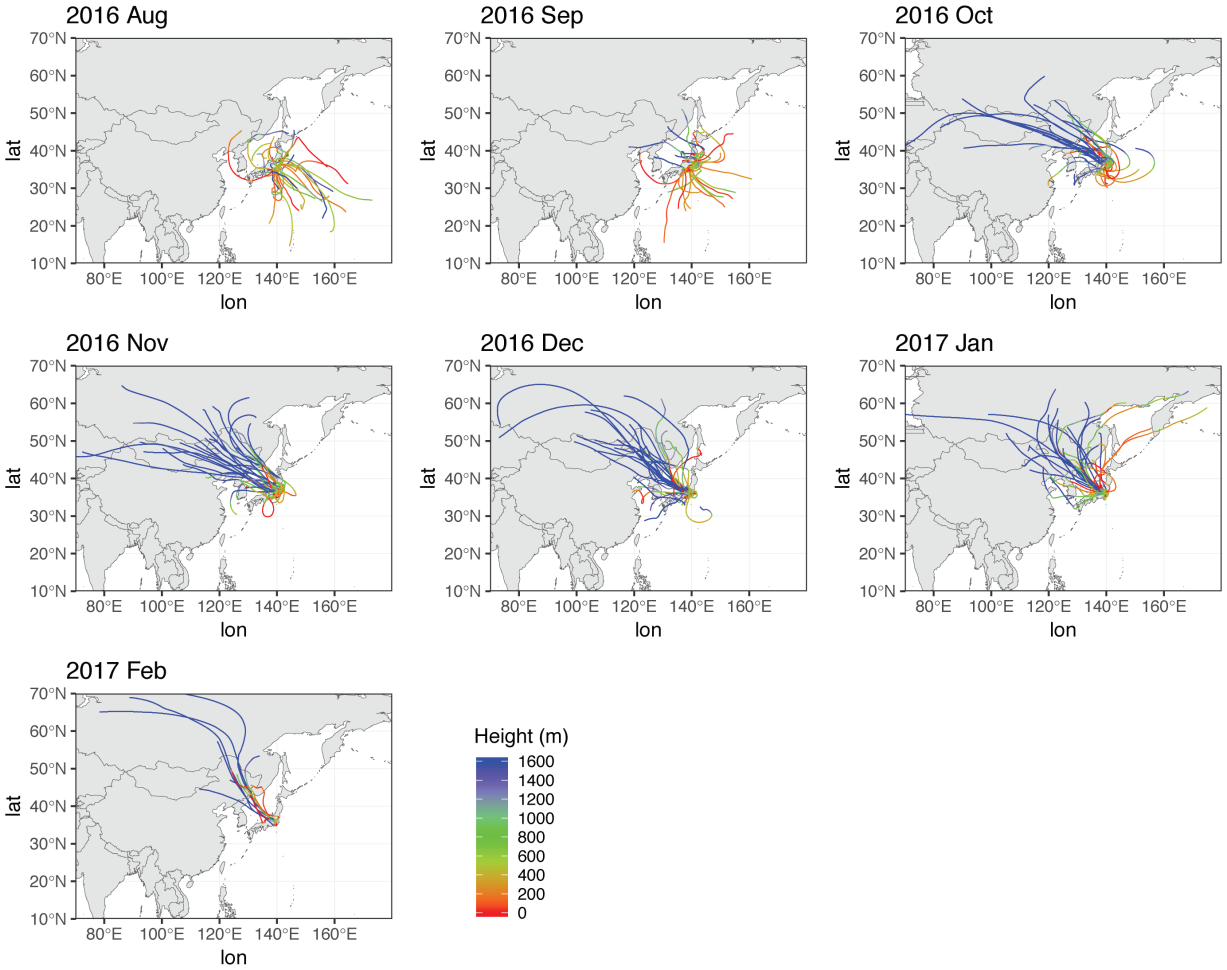
72 h HYSPLIT back trajectories during sampling period. Height above 1,600 m is shown in blue.

All meteorological data taken by the Automated Meteorological Data Acquisition System (AMeDAS), which is managed by the Japan Meteorological Agency (JMA), are available from http://www.data.jma.go.jp/obd/stats/etrn/index.php. Air temperature, wind speed, sunshine duration (total duration of solar radiation above 0.12 kW m^−2^), and humidity are taken at the “Edogawa rinkai” station and precipitation measurements were taken at the “Tokyo” station (Supplementary Table S1). Wave height data in Tokyo Bay, which is managed by the Bureau of Port and Harbor, Tokyo Metropolitan Government, are available from http://www.kouwan.metro.tokyo.jp/yakuwari/choui/kako1-index.html. All daily data were transformed to 2–3 day averages to correspond to sampling durations (Supplementary Table S2, Figure 1). The trajectories of air masses arriving at the sampling location were calculated by HYSPLIT (Cohen et al., 2015) using data from the GDAS 0.5 model (lat = 35.7101, lon = 139.8107, height = 458, duration = 72) and were started from 12:00 (UTC) of each day (Figure 2). All samples were categorized as either continental, meaning of Eurasian continent origin, or Pacific, based on their latitude/longitude location 72 h prior to arriving at Skytree.

## Results and Discussion

### Long-Range Effects of Air Mass Shifts Upon Bacterial Composition

Bacteria, which have round shapes and typical diameters/lengths of ~1–2 μm, are readily emitted from surfaces under certain conditions, such as by wind, mechanical disturbances, and bubble bursting, and may have aerial residence times from seconds to months (Burrows et al., 2009; Arnold et al., 2016), during which time they may be transported over great distances before their removal by both wet and dry deposition (Prospero et al., 2005; Barberán et al., 2014; Maki et al., 2017a). On non-dusty days, bacterial concentrations near ground level (10 m a.g.l.) are 10–100 times higher than in air at higher levels (Maki et al., 2017a), indicating that near-ground levels are strongly influenced by local sources. The average daytime atmospheric boundary layer height in Tokyo generally exceeds 500 m, even in wintertime, but is generally just below 500 m at nighttime over all seasons (Dien et al., 2017). Therefore, the Tokyo Skytree sampling site would likely sample boundary layer air primarily during daytime, and air from the residual layer and potentially the lower free troposphere during nighttime. Since samples were obtained continuously over several days, the deposited particulate matter in any one sample will be derived from this mix of air masses.

**Figure 3:**
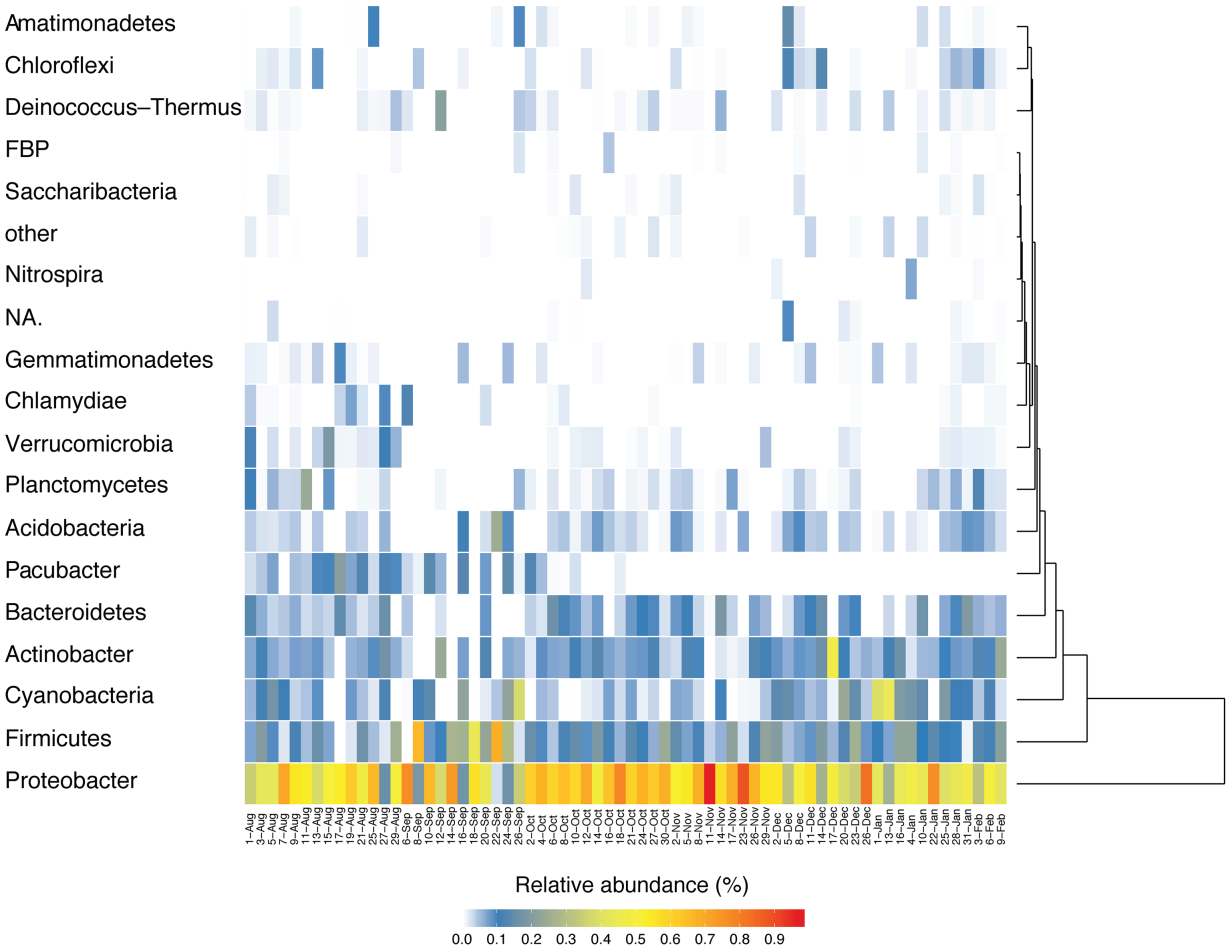
Seasonal change of bacteria at the phylum level.

In this study, an average of 5,862 sequences per sample (maximum: 19,913, minimum: 1,068) were used for analyses (Supplementary Table S2). Adequate coverage is required for reliable estimation by the indices (Hill et al., 2003), and indeed the depth of sequencing was enough for all three to stabilize (by 1,000 sequences; Supplementary Figure S2). Temporal variation of phylum *Proteobacteria* (mean 51.4% of relative abundances) was by far the most common followed by *Firmicutes* (13.6%), *Cyanobacteria* (7.9%), *Actinobacteria* (7.7%), *Bacteroidetes* (5.2%), and *Acidobacteria* (3.0%) (Figure 3). ANOSIM analyses on unweighted UniFrac distances (Supplementary Figure S3) clearly clustered into Pacific and continental sources (ANOSIM Global *R* = 0.337, *p* < 0.001: Supplementary Table S3), as predicted using HYSPLIT back trajectories.

Of all the phyla, only *Parcubacteria* (2.6%) exhibited seasonal changes, accounting for a larger fraction during August and September. A finer resolution of taxonomy (Supplementary Figure S2) shows that *Paraburkholderia* (mean 6.3%) had the highest mean relative abundance throughout the entire sampling period, followed by *Sphingomonas* (3.5%), *Chroococcidiopsis* (3.3%), and *Bacillus* (3.2%). Relative abundances of some genera were significantly (*p* < 0.05, Mann-Whitney) different in early October (Supplementary Table S4), when the HYSPLIT model indicated that the source region was shifted from Pacific to continental between October 2nd and 4th. At the genus level, the relative abundance of *Paraburkholderia* is significantly higher in the continental period, and that of an unidentified genus in the phylum *Parcubacteria* was higher in Pacific period (Supplementary Figure S4). Therefore, these genera are presumed to have originated from non-local sources, indicating that synoptic air mass movement influenced bacterial distributions.

To obtain more detail of these potentially long-range-transported bacteria, we investigated their ASV level taxonomy. Six ASVs (mostly members of the *Parcubacteria*) out of a total of 3,257 ASVs had significant differences in their abundances (*p* < 0.001, Mann-Whitney) between Pacific and continental periods (Supplementary Table S4, Supplementary Figure S5). The estimated sources of five of these ASVs (all the *Parcubacteria* and an *Opitutus* sp. from the *Verrucomicrobia*) that were abundant in the Pacific period, and hence were expected to be pelagic, were actually likely from freshwater environments according to their closest matches using BLAST searches (95.1–99.8% identity: Supplementary Table S5) and metametaDB analysis (25–78% MHI: Supplementary Figure S6). The sixth ASV (*Paraburkholderia* sp.) that was abundant in the period affected by continental air, and is mostly from soils (Supplementary Figure S6).

Their non-oceanic origin is also supported by the absence of ocean bacteria, such as bacteria belonging to the SAR group or typical marine groups (e.g., *Oceanospirillales*). Oceanospirillales are known to be readily aerosolized and carried long distances, since it was recorded in rainfall in the central Pyrenees mountains (Spain) by Cáliz et al. (2018), and at the Mt. Bachelor Observatory in North America (2.8 km above sea level) by Smith et al. (2013). This group seems to readily aerosolize and be transported long distances.

Thus, despite the HYSPLIT results, BLAST and metametaDB analysis for *Parcubacteria* and *Opitutus* indicate minimal inputs from the Pacific Ocean. This can be explained by a combination of low emissions from the ocean surface and higher inputs from regional freshwater. In previous studies, similarly lower contributions of ocean bacteria were reported. For example, the number of cultivable bacteria found in near-surface air during non-dust events over the ocean is generally very low (Prospero et al., 2005; Griffin et al., 2006), and the occurrence of marine bacteria in the near-surface air of a coastal city in Greece was found to be rare (Genitsaris et al., 2017).

One of the six ASVs, belonging to the genus *Paraburkholderia* (ASV137) and abundant in the continental period, was indicated to be a soil bacterium based on closest relative matches in BLAST searches (100% of identity: Supplementary Table S5) and metametaDB analysis (44% MHI: Supplementary Figure S6). Exactly the same sequences have been found in soil samples in many countries including China (e.g., KU323602, KX351056, KY427125). Since these were transported by westerly winds, which prevailed in the later sampling period when air masses mostly came from the Eurasian Continent, this ASV possibly originates from Chinese soil. Although some bacteria are known to be transported to Japan from arid regions in China during Asian dust events in March and April in Japan (Iwasaka et al., 2009), the genus *Paraburkholderia* (and its higher taxonomy order *Burkholderiales*) was not found in ground observations during Asian dust days (Park et al., 2016), in the upper Asian dust layer (Maki et al., 2017a) or in dust coming from the Gobi Desert, one of their potential source areas (Maki et al., 2017b). Therefore, ASV137 is not likely to be of Asian dust origin. Furthermore, *Bacillus subtilis*, which is common in Asian dust studies (Maki et al., 2010, 2017a), was found only once over the entire sampling period. Some *Bacillus* ASVs are relatively similar to uncultured bacteria from the Taklimakan Desert (AB696509 and AB696498); however, these were not perfectly matched by BLAST (ASV399: 99.1%, ASV6088: 98.6%, ASV1582: 98.1%). While six ASVs had long-range origins, the majority of ASVs were of local origin, and therefore were influenced by local meteorological conditions.

### Local Meteorological Effects Upon Sources of Bacteria

**Figure 4:**
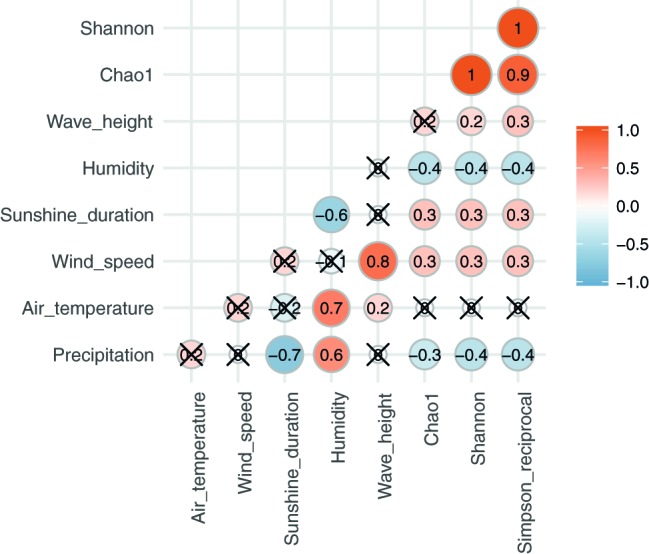
Spearman’s correlation between alpha diversities and meteorological and ocean factors. A red/orange circle shows positive correlation and a blue circle shows negative correlation. A cross “X” on the circle indicates no significance (*p* < 0.05).

Alpha diversities were relatively higher in August, October, and at the end of January and in the beginning of February. In contrast, there was also an extended period of consistently low alpha diversity during September (Supplementary Table S1, Figure 1). Correlation coefficients with the six local meteorological and ocean factors (Figure 4) show that, among meteorological and ocean factors, humidity and precipitation had the most significant correlations (both negative) with all diversity measures, while wind speed and sunshine duration had significant positive correlations with the alpha diversities. The same effects of local meteorology were observed in the PERMANOVA test of beta diversity on the effect of meteorological data (PERMANOVA, *p* < 0.005, Supplementary Table S6). While many previous studies did not analyze the cross-correlated meteorological variables (Burrows et al., 2009), multivariate statistics enable these relationships to be revealed (Bowers et al., 2013). We found that both simple correlations and multivariate analyses showed the same relationships, with humidity and wind speed being effective meteorological factors correlated with bacterial composition and diversity.

The effect of relative humidity changes on the composition of the airborne bacterial community has been reported previously. For example, canonical correspondence analyses of bacterial community structure in urban bioaerosols in Italy showed that both relative humidity and wind speed affected airborne bacterial community structure (Gandolfi et al., 2015), and a study in western Oregon, U.S.A, showed that the concentrations of airborne bacteria were positively correlated with temperature but negatively correlated with relative humidity (Tong and Lighthart, 2000). In the present study, relative humidity increased during precipitation events and remained high, before decreasing with an improvement in the weather (Figure 1). Relative humidity was especially high in late August – September, when it was strongly affected by a passing typhoon and an autumn rain event; alpha diversities were, correspondingly, low in the same period. High humidity tends to keep the surface of the ground wet, and the bonding force of surface tension will keep particles attached to the surface. This binding effect reduces with drying (Jones and Harrison, 2004; Šantl-Temkiv et al., 2018).

**Figure 5:**
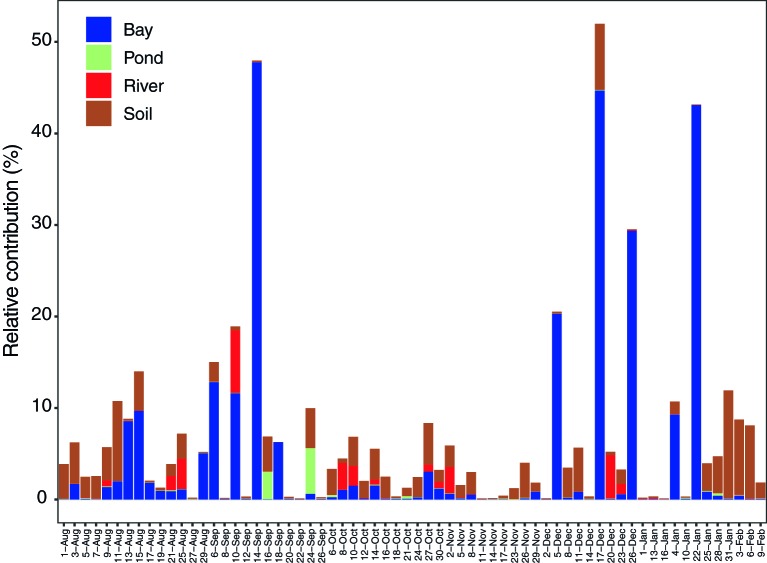
Seasonal change of estimated contribution from potential source types (bay, soil, pond, and river) by source tracking analysis.

**Figure 6:**
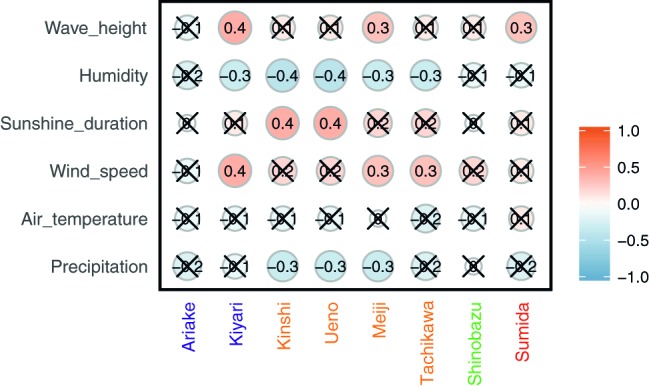
Spearman’s correlation between estimated source contribution and meteorological factors and wave height in Tokyo Bay. A red/orange circle shows positive correlation and a blue circle shows negative correlation. Cross “X” on the circle indicates no significance (*p* < 0.05).

Some potential source areas were also affected by humidity. Sourcetracker2 analysis shows the estimated contributions to Tokyo Skytree air samples from each potential source (Figure 5 for each category, Supplementary Figure S7). Total percentage contributions from the tested sources had a maximum of 52%, a minimum of 0.1%, and a mean of 6.8%. Correlations between source contributions and environmental factors (Figure 6) show that, among the six environmental factors, humidity had a significant negative correlation for all soil sites (Kiyari, Ueno, Meiji and Tachikawa). This correlation was highest in the middle of winter, the driest season during the sampling period (Figures 1, 6).

Precipitation, which obviously strongly affects the humidity and surface bonding forces, was, however, not found to have a consistent or strong relationship with airborne bacterial diversity (correlation coefficient: −0.3 to −0.4) and composition (Psuedo-F: 1.48, *p* = 0.023). While alpha diversities tended to decrease during precipitation events (Supplementary Figure S8: blue broken lines), exceptions occurred during heavy precipitation events resulting from a typhoon that set historical precipitation records and during some stationary fronts (Supplementary Figure S8: red broken lines). The common expectation, and what occurred during the majority of lighter precipitation events, is that airborne bacterial communities would be reduced by precipitation due to scavenging (wet deposition) (Tong and Lighthart, 2000; Yue et al., 2016) and wetting of the soil surface. However, alpha diversities slightly increased during the historical heavy rains on Aug. 21–22, Sep. 12–13, and Sep. 22–23 (Supplementary Figure S8: red broken line). For example, rain rates of 107.5 mm h^−1^ were reported in Ome (30 km west from the site) on Aug. 22 during typhoon passage, and 63.5 mm h^−1^ in Yokoshibahikari (80 km east) under a stationary front condition. Diversity increase during such heavy rain events may be explained by bioaerosol generation caused by the impact of large rain drops on plant, soil, and built surfaces (Tong and Lighthart, 2000; Wang et al., 2016; Joung et al., 2017; Kim et al., 2019), and in this case potential sources would be near the sampling site (e.g., even the outside walkway and wall of the tower).

Wind speed was positively correlated with airborne microbial concentrations in previous studies (Bovallius et al., 1978; Lighthart, 1997; Mouli et al., 2005). As well, dynamic water surfaces enhance aerosol generation into the atmosphere (Aller et al., 2005; Dueker et al., 2011; DeLeon-Rodriguez et al., 2013; Maki et al., 2017a; Cáliz et al., 2018). Wind speed is highest in late August, and was significantly correlated with wave height in Tokyo Bay (Figure 1). High wind speeds over the bay’s surface resulted in rougher waters, which would be expected to correlate with more aerosol generation *via* the bubble bursting process (Burrows et al., 2009) during wave-breaking, especially at the shore. Among the three potential sources, the bay sample had the most significant positive correlation of source contribution with wind speed and wave height. Wind speed was also weakly correlated with two of the soil sites (Meiji and Tachikawa), suggesting that dispersal of bacteria from the soil surface was enhanced by stronger winds.

Many previous studies showed temperature, which follows seasonal cycles in temperate regions, is also a factor controlling airborne bacterial emissions and community composition (Tong and Lighthart, 2000; Jones and Harrison, 2004; Bowers et al., 2013; Genitsaris et al., 2017). In contrast, temperature was not found to be a significant factor in this study.

The effects of local meteorological conditions upon airborne microorganisms are complicated, because recent studies have suggested that airborne bacterial communities are more affected by the nature of local sources than by changes in local meteorological conditions (Bowers et al., 2011a, 2013). This study, and other works, found raised relative humidity and rainfall were the principal drivers of primary bioaerosols and ice nucleating particle emissions (Huffman et al., 2013; Prenni et al., 2013; Tobo et al., 2013; Wright et al., 2014; Mason et al., 2015). Bacterial diversity and community structure are clearly influenced by both local ecotypes and meteorological factors.

In this study, air mass shifts detected by HYSPLIT analysis indicated limited long-range contributions. Estimated contributions from local sources, from seawater in Tokyo Bay and soil, were consistently found and predominated over other known sources over the sampling period (Figure 5). Since the samples used for source estimation were limited in type, number of sites, and season, our reference samples only explained, on average, <6.9% of the total relative contribution from multiple sources. This implies that we should include more diverse potential source types in future studies, such as plant surfaces (Šantl-Temkiv et al., 2012) and sites with exposed animal (cow, dog, and human) feces (Bowers et al., 2013). Adding more, and more varied, reference samples will likely enable more accurate and comprehensive source estimations.

Since a high proportion of airborne bacteria has been shown to remain viable after continental scale long-range transport (Hara and Zhang, 2012), deposition of airborne bacterial communities plays a crucial role in the local microbiome (Barberán et al., 2014; Honeyman et al., 2018). All Tokyo sites would be influenced and perhaps modified by the deposition of such airborne bacteria. However, deposited microbes will only persist if their new niche is favorable. For example, the genus *Paraburkholderia*, which dominated in the Continental period, was found only in one out of four soil sites (at NIPR).

In order to obtain enough DNA for analysis, we obtained samples that were integrated over 2–3 days. However, shorter sampling intervals may be helpful for understanding the effect of radical meteorological changes upon microbial communities, such as heavy rain enhancement of microbial emissions versus precipitation scavenging in other rain events. Recently, much higher flow rate and lower cost sampling devices have been proposed (Šantl-Temkiv et al., 2017) for use in the field (Šantl-Temkiv et al., 2018). Such capabilities may offer the ability to understand not only hourly changes of specific microbes, but also offer sufficient sample sizes to permit metagenomic analyses of potential microbiome function.

## Conclusions

Consecutive 48–72 h samples from the tallest tower in Japan, combined with NGS analyses, were used to study airborne microbial communities from summer to winter. In this study, air mass shifts detected by HYSPLIT analysis indicated limited long-range effects on microbial populations. Our results showed only a limited number of ASVs that could potentially be associated with long-distance transport of bacteria. Further, relative abundances of most of the ASVs were not affected by abrupt air mass changes, indicating they were likely to have been from local sources. Source tracking analysis indicated that local inputs, from soil and the seawater in Tokyo Bay, were consistently found and were the predominant identified sources over the study period. Correlation and PERMANOVA analyses indicated that humidity and wind speed were key factors affecting bacterial alpha and beta diversity, and hence, these appear to be the controlling factors on emissions of bacteria from bay seawater and soil around the Tokyo Skytree. The combination of NGS, reliable metadata, and powerful statistical tools allowed us to study the airborne microbiome near the Tokyo Skytree with unprecedented resolution, and the accumulation of such knowledge from many environments across the world could provide a more comprehensive understanding of factors determining local, regional, and global airborne microbiomes.

## Data Availability

Publicly available datasets were analyzed in this study. This data can be found here: https://doi.org/10.6084/m9.figshare.7772864.

## Author Contributions

JU designed and managed the research. JU, YT, YU, and RM set up the laboratory on the Tokyo Skytree. JU was responsible for sampling, with support from YT and YU. JU performed the bioinformatics and statistical analyses of the sequencing data. JU, YT, TH, PD, and SK wrote the manuscript. All authors read, edited, and approved the final manuscript.

### Conflict of Interest Statement

The authors declare that the research was conducted in the absence of any commercial or financial relationships that could be construed as a potential conflict of interest.

## Footnotes

^1^https://support.illumina.com/content/dam/illumina-support/documents/documentation/chemistry_documentation/16s/16s-metagenomic-library-prep-guide-15044223-b.pdf

^2^https://www.geneious.com/

^3^https://github.com/biota/sourcetracker2

^4^https://www.xlstat.com
