# Supplementary material for: Seasonal Changes of Airborne Bacterial Communities Over Tokyo and Influence of Local Meteorology
Source: Front Microbiol. 2019 Jul 16;10:1572. doi: 10.3389/fmicb.2019.01572 (PMC6646838; doi:10.3389/fmicb.2019.01572)
Supplement: Supplementary file 1 [file Data_Sheet_1.PDF]

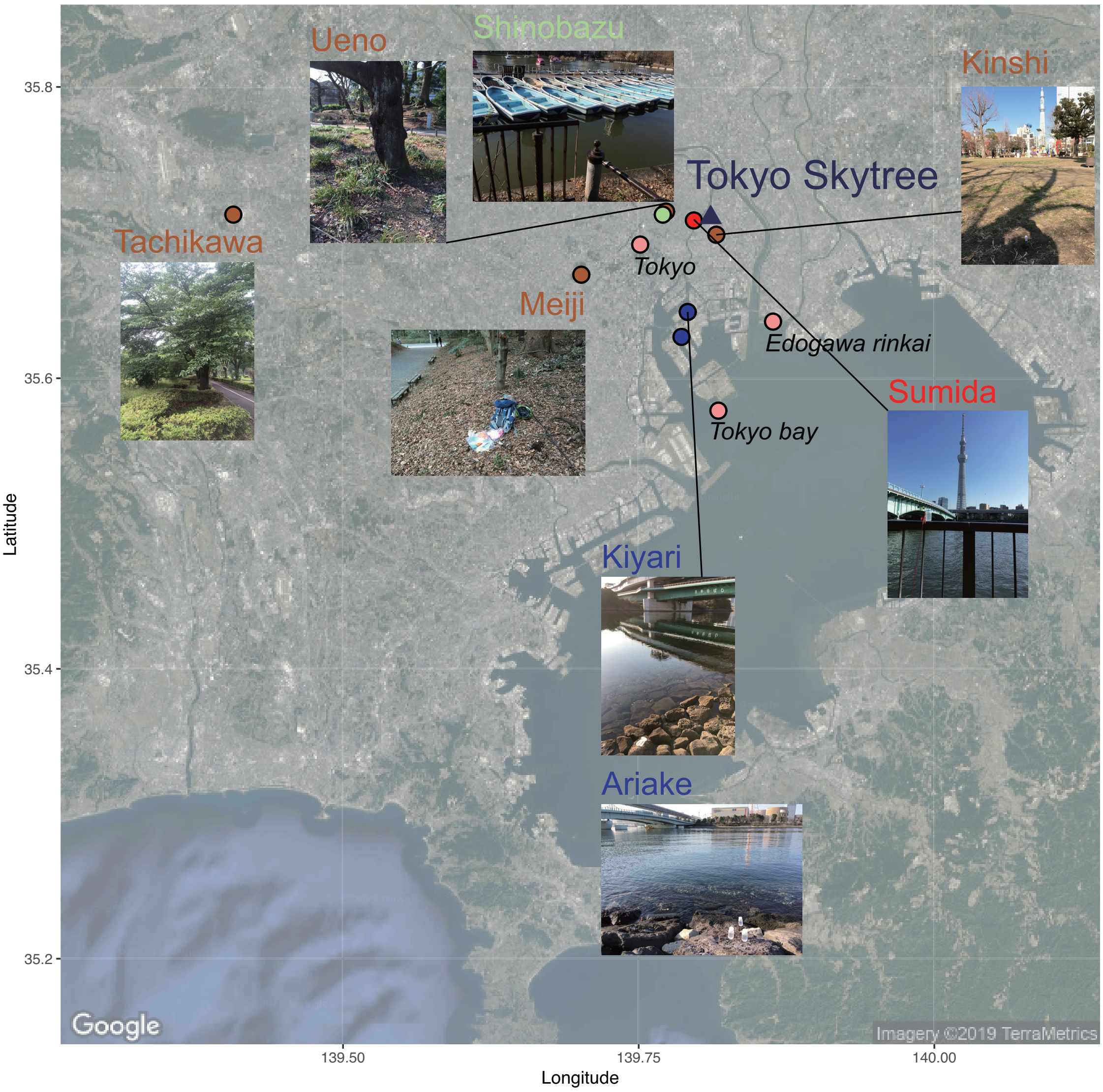

SI Fig. 1: Location of Tokyo Skytree (blue triangle), sampling sites for reference (blue circle: bay, brow circle: soil, red circle: river and green circle: pond) and meteorological and oceanic observation sites (pink circle). More detailed information is available in SI Table.1.

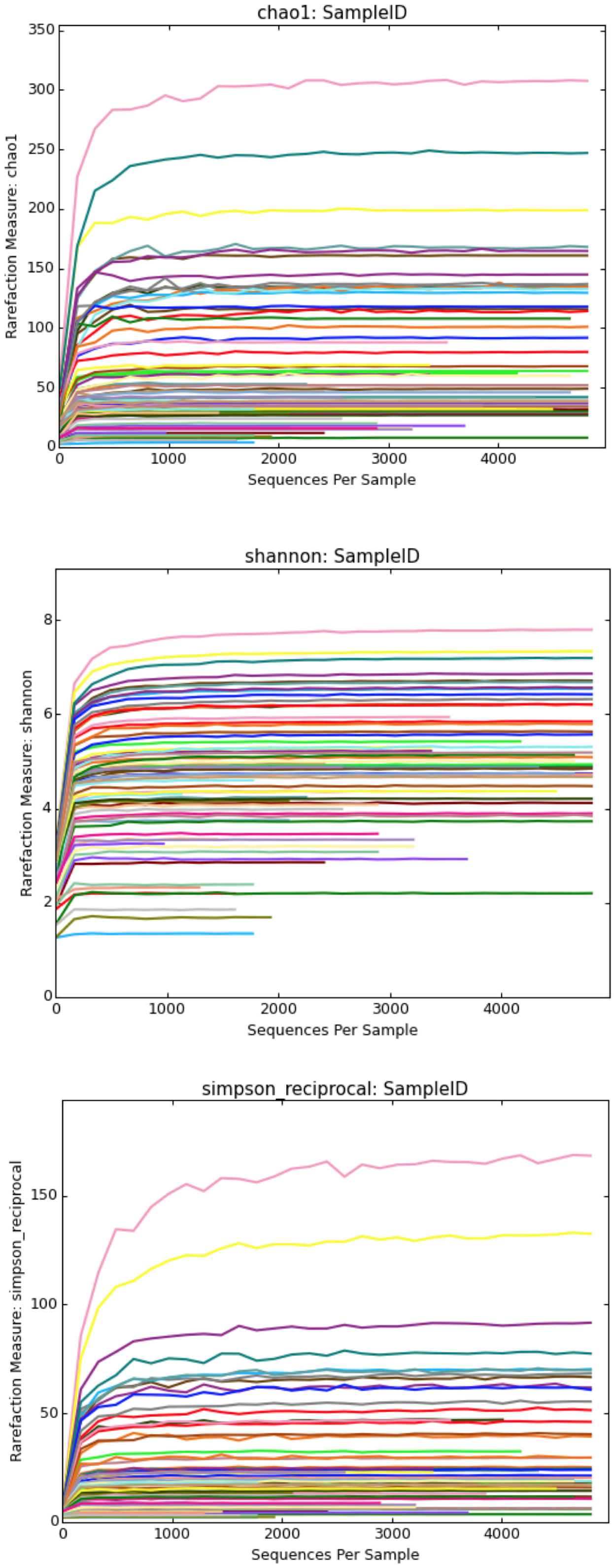

SI Fig. 2: Rarefaction curves of three alpha diversity methods.

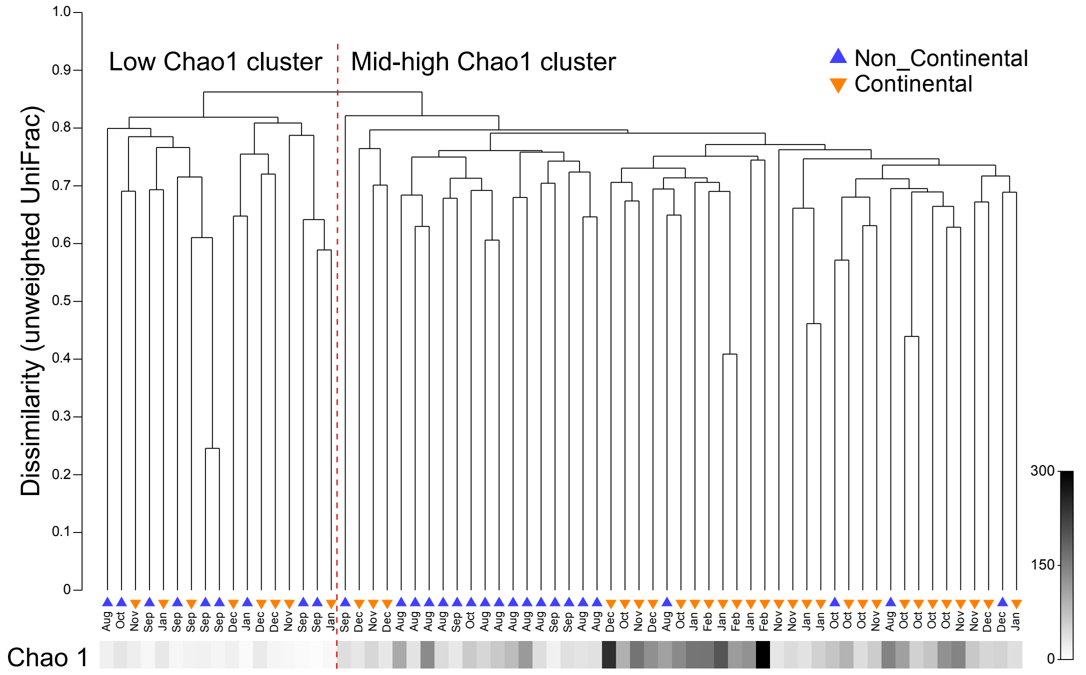

SI Fig. 3: Unweighted Pair Group Method with Arithmetic Mean (UPGMA) dendrogram of dissimilarity by unweighted-UniFrac, shown with changes of Chao1 (gray bar in the bottom).

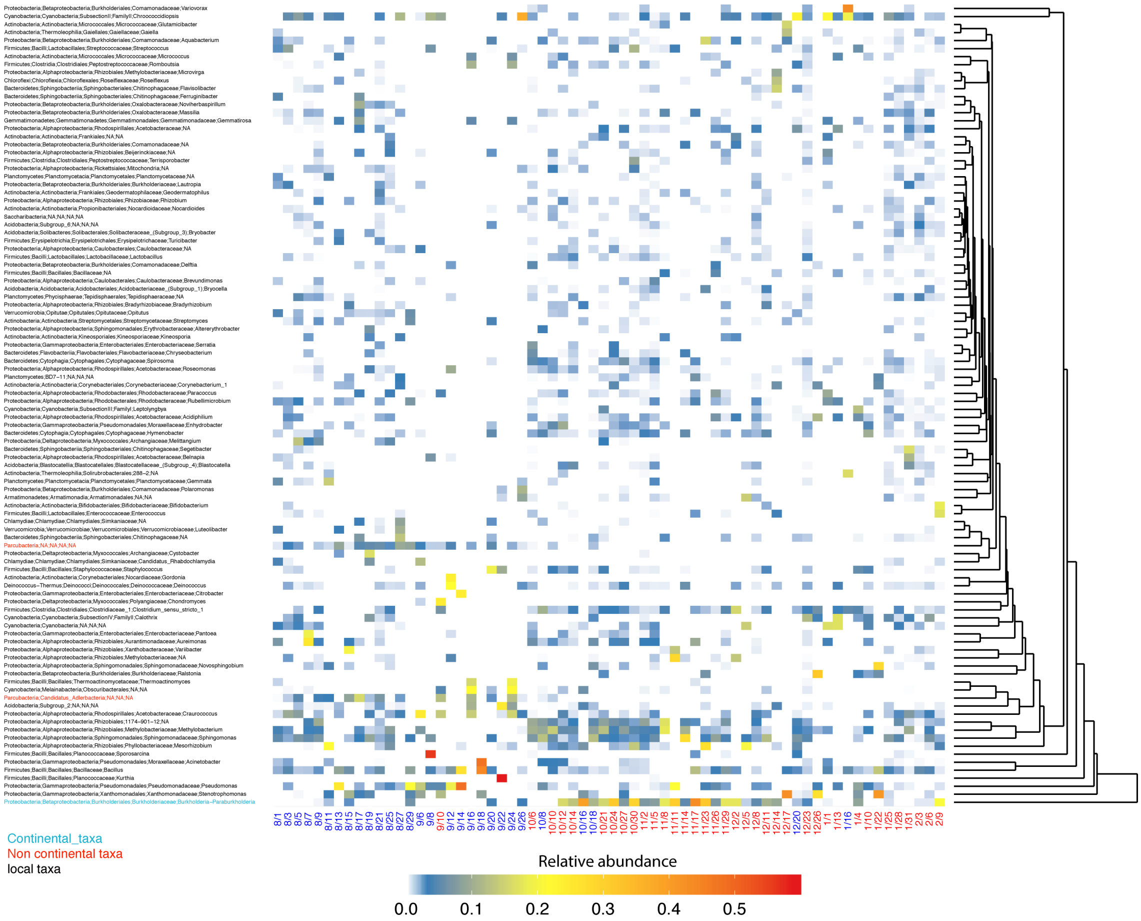

SI Fig. 4: Seasonal change of bacteria at the genus level.

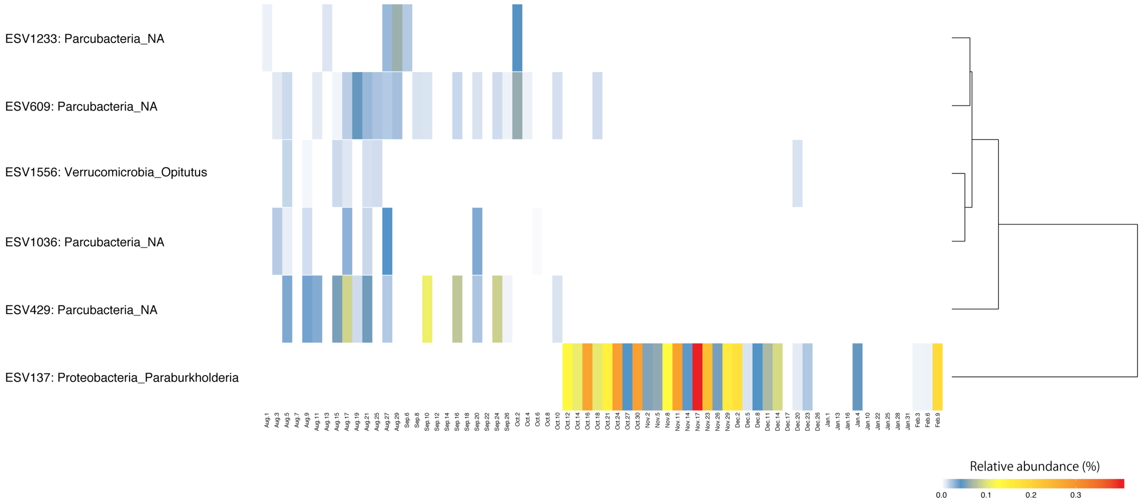

SI Fig. 5: Seasonal change of 6 ASVs, which were strongly affected by air mass change in early October.

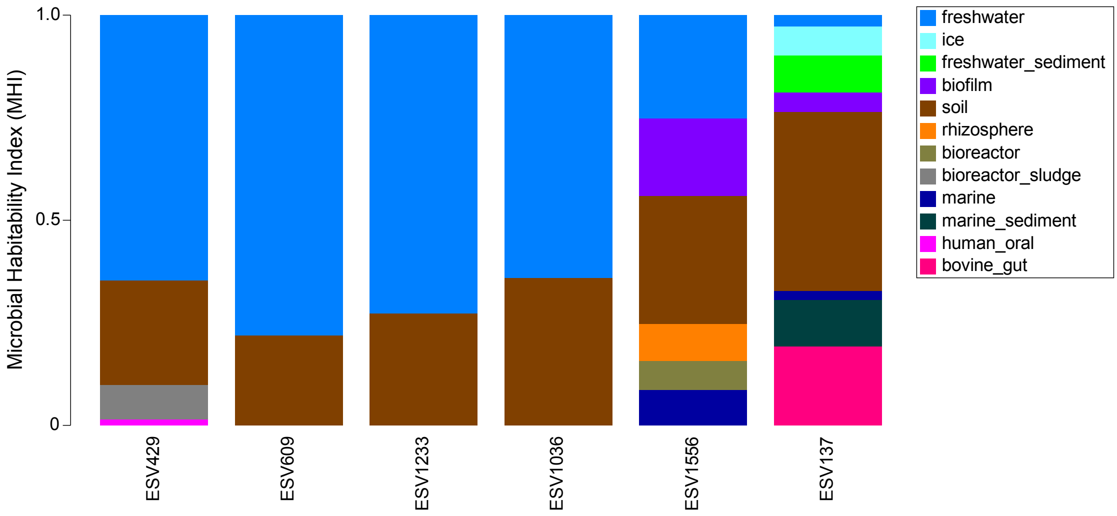

SI Fig. 6: Microbial habitability index for 6 ASVs estimated by metametaDB, which infers the possibility to find microbes in each environment.

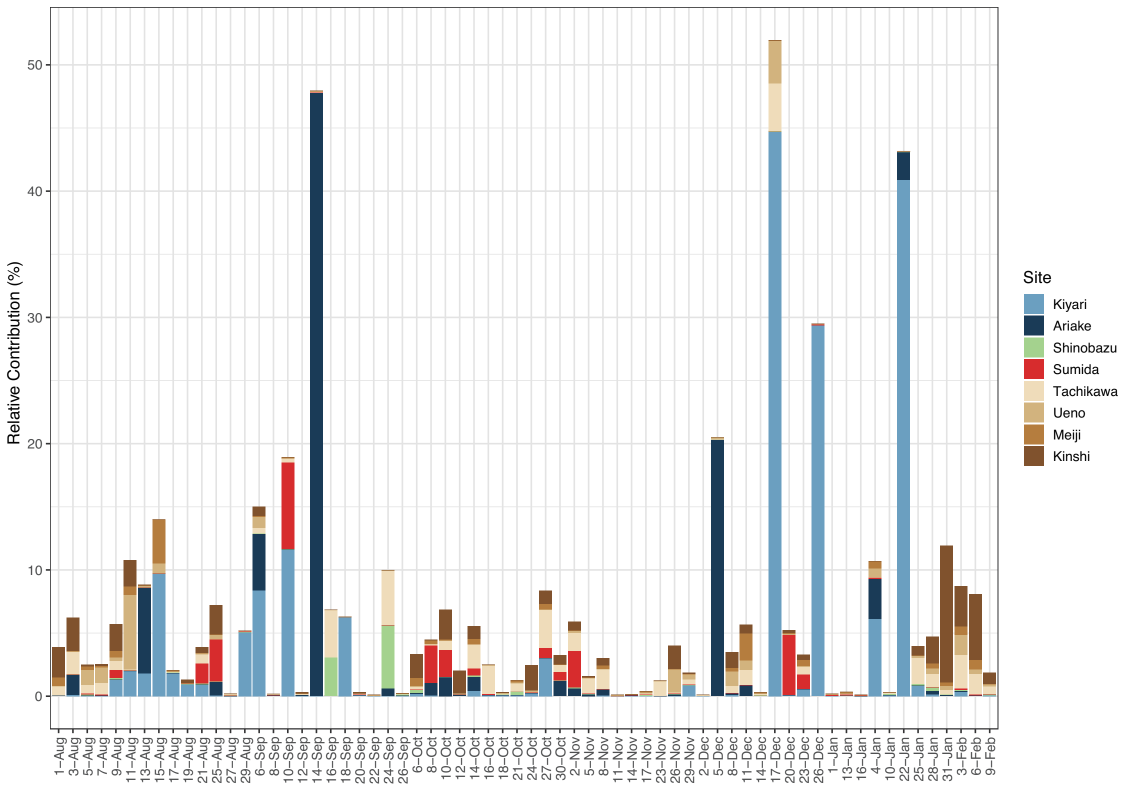

SI Fig. 7: Seasonal change of estimated contribution from 8 potential sites in 4 categories (bay, soil, pond and river) by source tracking analysis.

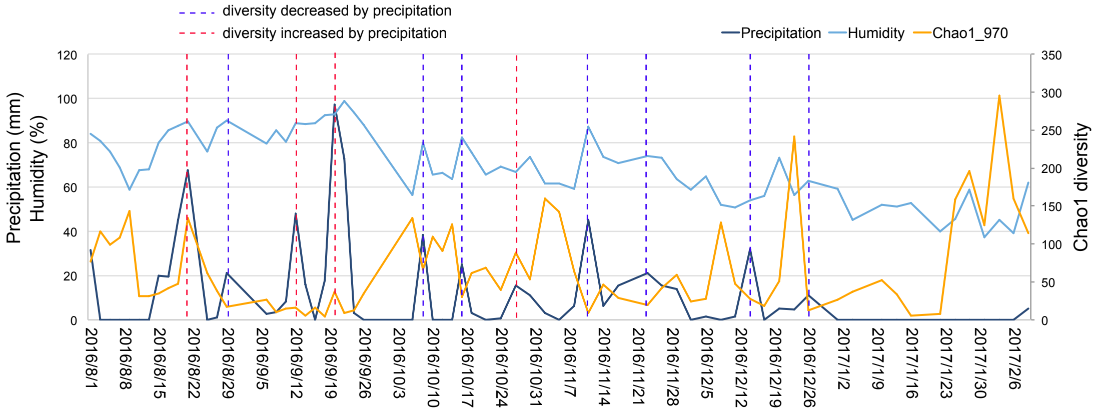

SI Fig.8: Changes of Chao 1 index and potentially related meteorology factors (precipitation and humidity). Blue broken lines show sampling periods when Chao 1 diversity decreased during precipitation in normal rain events. Red broken lines show sampling periods when Chao 1 diversity increased during precipitation, primarily occurring during a historically heavy rain event.
